# Tandem straight fenestrated clipping for posterior communicating artery aneurysm with fetal-type posterior cerebral artery

**DOI:** 10.1007/s00701-026-06938-9

**Published:** 2026-06-08

**Authors:** Kristy Latour, Marco Maria Fontanella, Bin Xu, Feng Xu

**Affiliations:** 1 Neurosurgery Unit, Department of Medical and Surgical Specialties, Radiological Sciences and Public Health, University of Brescia, Brescia, Italy; 2 Department of Neurosurgery, Huashan Hospital, Shanghai Medical College, Fudan University, Shanghai, China; 3 National Center for Neurological Disorders, Shanghai, China; 4 Shanghai Key Laboratory of Brain Function and Restoration and Neural Regeneration, Shanghai, China; 5 Neurosurgical Institute of Fudan University, Shanghai, China; 6 Shanghai Clinical Medical Center of Neurosurgery, Shanghai, China

**Keywords:** Posterior communicating artery aneurysm, Fetal-type posterior cerebral artery, Microsurgical clipping, Vascular neurosurgery, Intraoperative neuromonitoring

## Abstract

**Background:**

In 20% of patients, a dominant posterior communicating artery (PCoA) supplies the occipital lobe owing to a hypoplastic P1 segment of the posterior cerebral artery (PCA).

**Method:**

We present a reconstructive technique involving straight fenestrated clips applied in tandem for the exclusion of a postero-laterally projecting, wide-necked PCoA aneurysm with limited dome height and associated with a fetal-type PCA.

**Conclusion:**

This configuration progressively reconstructs the supraclinoid internal carotid artery-PCoA junction, completely excludes the aneurysm neck and avoids clip slippage. Continuous intraoperative neuromonitoring supports safe flow control and patency of the perforators and anterior choroidal artery.

**Supplementary Information:**

The online version contains supplementary material available at 10.1007/s00701-026-06938-9.

## Relevant surgical anatomy

The posterior communicating artery (PCoA) arises from the posterior wall of the supraclinoid internal carotid artery (ICA) and travels posteriorly to the posterior cerebral artery (PCA) at the P1-P2 junction, along the superior surface of the oculomotor nerve [8]. In 40% of patients, up to 14 perforators branch from the PCoA and the short segment of the ICA between the PCoA and the anterior choroidal artery, supplying the oculomotor nerve, cerebral crura, ventral thalamus, and caudate nucleus [2].

In 15 to 22% of patients, the posterior cerebral artery originates from a dominant PCoA and the P1 segment is hypoplastic. This anatomical variation, corresponding to the initial embryological pattern, is referred to as the fetal origin of the PCA (fPCA) [1].

In this configuration, the PCA receives its supply predominantly from the ICA rather than the vertebrobasilar system.

**Fig. 1:**
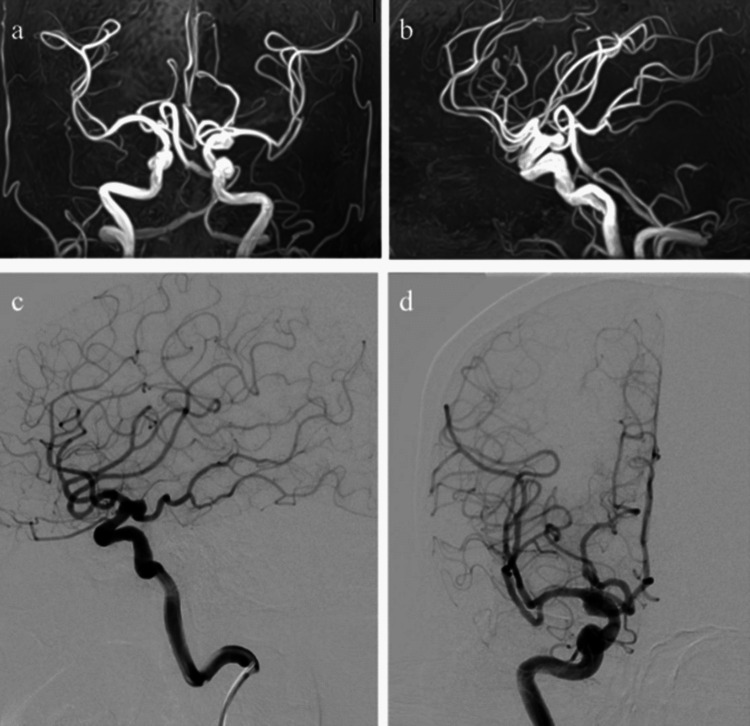
Pre-operative magnetic resonance angiography (MRA) in coronal (**a**) and sagittal (**b**) views and digital subtraction angiography (DSA) in lateral (**c**) and anteroposterior (**d**) view showing a wide-necked postero-laterally projecting saccular aneurysm of the right PCoA associated with fetal-type PCA and mild supraclinoid ICA stenosis

Aneurysms of the PCoA comprise approximately 20 to 25% of all intracranial aneurysms and commonly project posteriorly and postero-laterally [5]. We present a reconstructive clipping strategy for such aneurysm (Fig. 1), in which recognition of a fetal-type PCA variant is essential as its inadvertent compromise can lead to occipital lobe infarction.

## Description of the technique

Owing to postero-lateral projection of the aneurysm dome, the patient’s head was turned 60° contralaterally to visualize the aneurysm’s lateral profile. Through lateral supraorbital approach and minimal sylvian fissure dissection, the optic-carotid triangle was opened and the optic nerve identified, overlying the supraclinoid ICA at the point of proximal control. Sharp dissection proceeded laterally around an atherosclerotic supraclinoid ICA to its inferior surface and the distal posterior communicating artery was followed proximally to confirm its origin. The course of the PCoA, its perforating branches, and the anterior choroidal artery were clearly visualized. The aneurysm dome projected postero-laterally toward the tentorial edge, lying adjacent to the oculomotor nerve. Two critical points were identified: the interval between the PCoA origin and the proximal aneurysm neck, and the one between the anterior choroidal artery (AchA) and the distal aneurysm neck. The AChA was dissected free from the distal aneurysm neck.

**Fig. 2:**
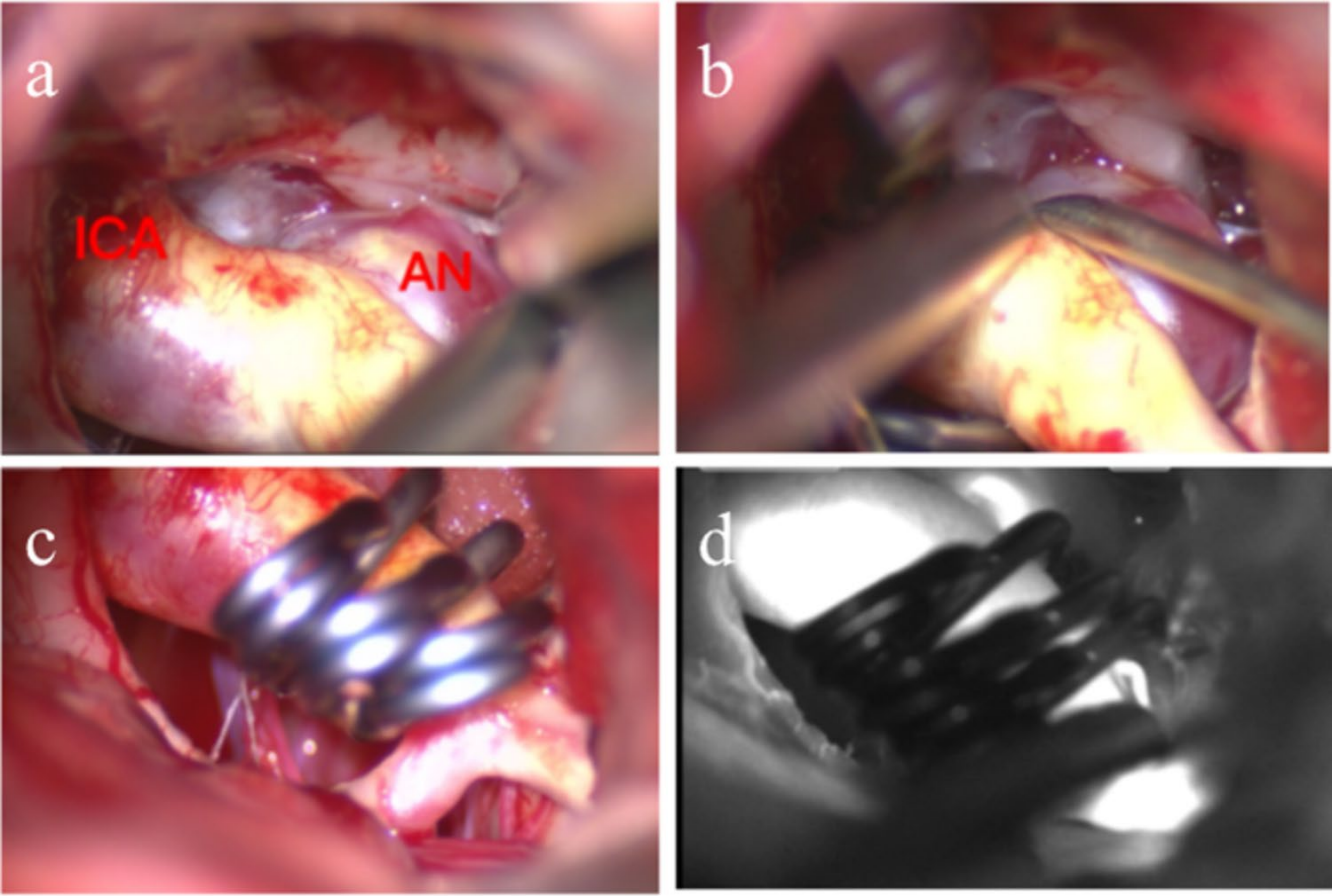
Intraoperative views. **a** exposure of the supraclinoid ICA and a wide aneurysm neck with limited dome height; **b** III cranial nerve and PCoA were identified; **c** tandem straight fenestrated clips were oriented parallel to the PCoA; **d** ICG confirmed complete aneurysm exclusion, preservation of the AchA and PCoA

**Fig. 3:**
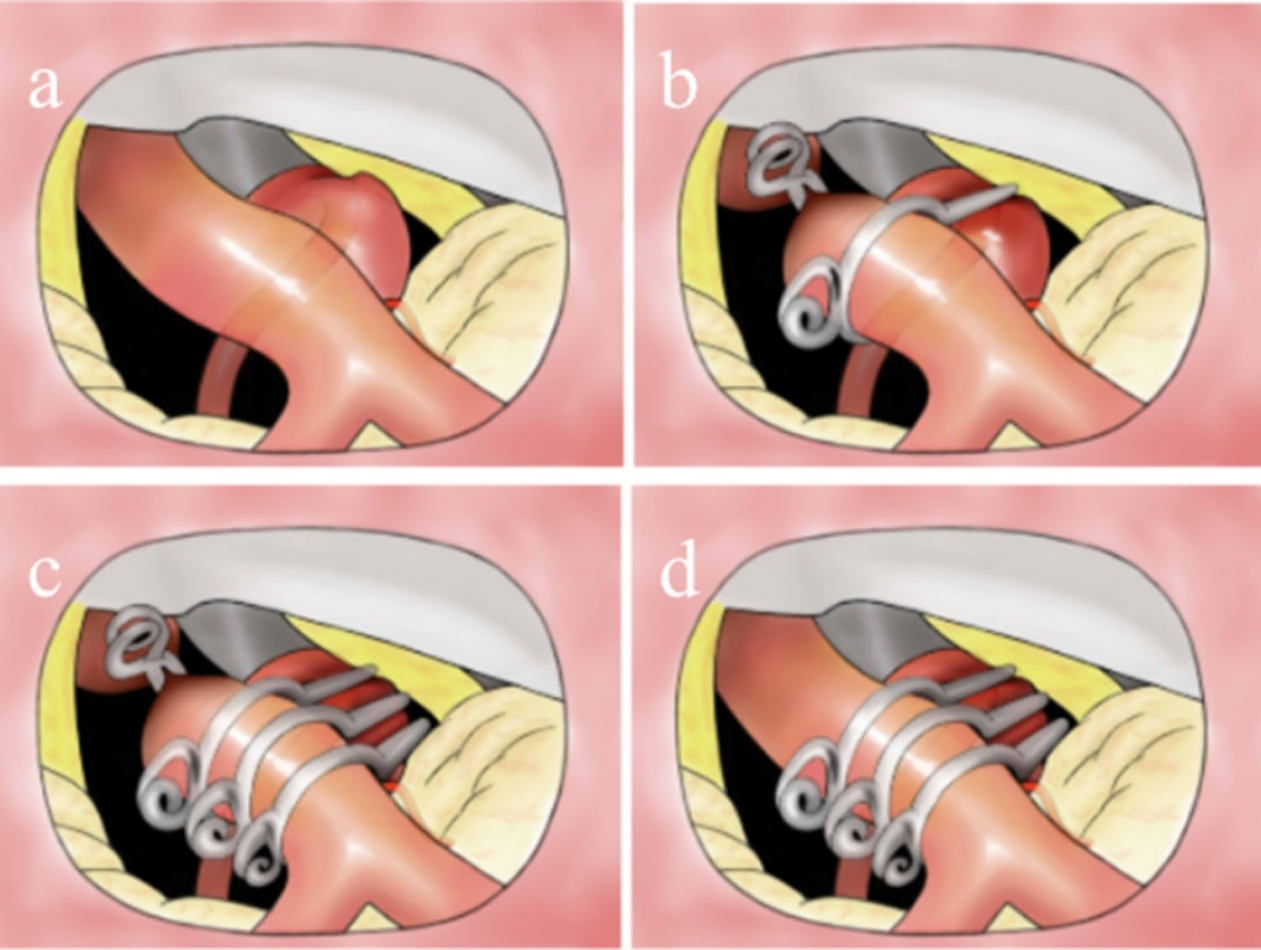
Stepwise illustration of vessel reconstruction using tandem straight fenestrated clips in a PCoA aneurysm. **a** wide-necked posterolaterally projecting aneurysm; **b** temporary proximal control on the supraclinoid ICA and application of a straight fenestrated clip parallel to the PCoA; **c** tandem placement of straight fenestrated clips to progressively reconstruct both the supraclinoid ICA and PCoA; **d** final clip configuration demonstrating complete neck exclusion, preservation of the supraclinoid ICA, PCoA and AchA

**Fig. 4:**
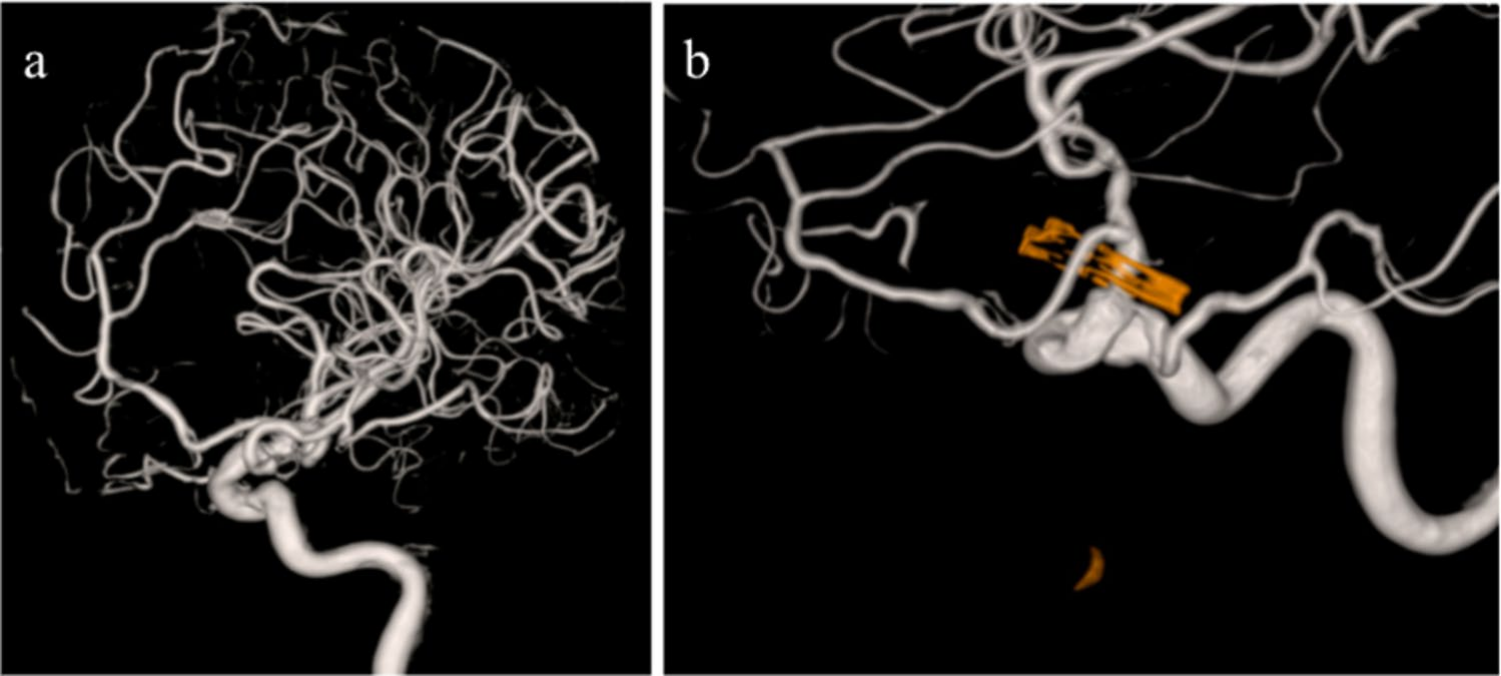
Angiographic follow-up in anteroposterior (**a**) and lateral (**b**) views at 52 months showed no evidence of aneurysm recurrence, with only minimal dilatation of the PCoA

Brief, intermittent temporary clipping of the supraclinoid ICA was performed under continuous intraoperative neuromonitoring for proximal control. When required to facilitate safe aneurysm dissection, additional temporary clips were applied selectively on A1 and PCoA to achieve brief segmental flow arrest (Video 1). Occlusion times were limited to less than 3 min in light of preoperative evidence of ICA stenosis and were guided by continuous intraoperative somatosensory evoked potentials (SSEP) and motor evoked potentials (MEP). Tandem straight fenestrated clips were applied parallel to the PCoA (Fig. 2). The clip blades reconstructed both the PCoA and the postero-lateral wall of the supraclinoid ICA, achieving complete obliteration of the wide-necked aneurysm. The inferomedial contour of the supraclinoid ICA was restored by careful alignment of the clip heels (Fig. 3). Doppler ultrasonography was used to confirm flow in the PCoA, and indocyanine green videoangiography (ICG) demonstrated preservation of perforator branch flow and the absence of a residual neck. At 52-month angiographic follow-up, no aneurysm recurrence was observed, and the patient was neurologically intact (Fig. 4).

## Indications

Small, unruptured PComA aneurysms can often be managed with radiological follow-up. However, in the present case, surgical treatment was favored due to the patient’s risk factors including hypertension and hyperlipidemia. In addition, high-resolution vessel wall MRI (HR-VW-MRI) demonstrated aneurysm wall enhancement, suggesting instability and the aneurysm showed irregular morphology with a daughter sac. Endovascular treatment was also considered less favorable: in wide-necked PCoA aneurysms with a fetal-type PCA and limited dome height, Y- or T-stent assisted coiling is technically demanding and carries a non-negligible risk of complications. On the other hand, flow diversion may be associated with lower rates of complete occlusion [6]. In this setting, tandem application of straight fenestrated clips parallel to the PCoA and guided by intra-operative neuromonitoring represented a safer and more effective option, minimizing the risks of clip slippage, intraoperative rupture and branch vessel stenosis.

## Limitations

PCoA aneurysms projecting medially, posteriorly, or superiorly may have the neck and fundus partially hidden by the anterior clinoid process and distal dural ring, and clinoidectomy can expand this triangular working corridor. Nonetheless, the tandem straight fenestrated clip strategy has important limitations: extensive ICA atherosclerosis may restrict safe mobilization and reduce tolerance to prolonged temporary clipping, while deep and narrow operative corridors may still compromise visualization and maneuverability, favoring alternative clip geometries or different treatment approaches. In addition, this technique may be less suitable in patients with poor collateral circulation or limited tolerance to temporary carotid occlusion.

## How to avoid complications

In the presence of a fetal-type PCA, parent vessel reconstruction and preservation of the PCoA is critical, as compromise may result in occipital infarction.

Straight fenestrated clips enable progressive reconstruction of the supraclinoid ICA-PCoA junction, with the fenestrations encircling the parent vessel. The blades of multiple clips gradually reconstruct the wide aneurysm neck, reducing the risk of branching artery stenosis or incomplete aneurysm obliteration. Sugita et al. advanced fenestrated clipping for parent vessel reconstruction by designing 24 different types of fenestrated clips with varying fenestration diameters [7]. To enable arterial wall reconstruction, Sugita introduced different blade geometry including short and long blades with either straight, angled or bayonet configurations.

In our case, straight blades were sufficient owing to an adequately wide optic-carotid corridor. Indeed, a 3 mm incision of the falciform ligament releases the optic-carotid tether, facilitates mobilization of the supraclinoid carotid artery and increases the working corridor without the need for routine anterior clinoidectomy. This must be done prior to dissection of the aneurysm dome in order to gain appropriate control for final dissection and for ease of management of potential intraoperative rupture [3, 4]

Intraoperative neurophysiological monitoring should be routinely used to conduct safe temporary clipping and to confirm patency of the parent vessel, PCoA, perforators and AchA.

## Specific information for the patient

The aim is to achieve complete aneurysm exclusion while protecting vessels’ patency. Safety of the reconstructive technique is supported by continuous intraoperative neuromonitoring.

## 10 key points summary

1. Anterior clinoidectomy is not routinely required for PcomA aneurysm clipping but is useful in selected complex cases to improve exposure of the aneurysm neck and adjacent ICA when standard exposure is insufficent.
2. PCoA aneurysms associated with a fetal-type PCA mandate a reconstructive clipping configuration.
3. Straight clips are commonly applied parallel to the PCoA to preserve its patency and avoid kinking.
4. Limited aneurysm dome height along with a markedly wide neck rendered the use of standard straight clips unsafe owing to increased risk of clip slippage and consequent intraoperative aneurysm rupture.
5. Although perpendicular fenestrated clips are frequently used for paraclinoid ICA aneurysm, this configuration was also deemed unsafe due to excessive clip length and risk of compromising the PCoA and the AchA.
6. Decompress first, then free the nerve: temporary clipping softens large PCoA aneurysms, allowing safe oculomotor nerve and clear clip-blade placement without nerve involvement.
7. Following temporary proximal control, tandem straight fenestrated clips can be applied parallel to the PCoA. This alignment progressively reconstructs both the supraclinoid ICA and the PCoA contour while excluding the wide aneurysm neck.
8. Intermittent temporary clipping of the ICA and short-duration flow arrest of the A1 and PCoA, when required, can facilitate safe aneurysm dissection.
9. Continuous intraoperative neurophysiological monitoring with somatosensory evoked potentials (SSEP) and motor evoked potentials (MEP) supports safe flow control and patency of perforators and the AchA.
10. Long-term follow-up angiography is essential for the identification of aneurysm recurrence.

## Supplementary Information

Below is the link to the electronic supplementary material.

ESM 1 Supplementary Material 1 (MP4 168 MB)

## Data Availability

No datasets were generated or analysed during the current study.

## Abbreviations

AchA: Anterior choroidal artery
DSA: Digital subtraction angiography
EEG: Electroencephalography
HR-VW-MRI: High-resolution vessel wall MRI
ICA: Internal carotid artery
ICG: Indocyanine green videoangiography
fPCA: Fetal-type PCA
FD: Flow diversion
MEP: Motor evoked potentials
MRA: Magnetic resonance angiography
PCA: Posterior cerebral artery
PCoA: Posterior communicating artery
SSEP: Somatosensory evoked potentials
